# The CD79α (HM47/A9) antibody is effective in distinguishing between primary hepatocellular carcinoma and primary intrahepatic cholangiocarcinoma

**DOI:** 10.3892/ol.2013.1163

**Published:** 2013-01-30

**Authors:** HAO LI, CONGYANG LI

**Affiliations:** Department of Pathology, People’s Liberation Army 152 Hospital, Pingdingshan, Henan 467000, P.R. China

**Keywords:** primary hepatocellular carcinoma, primary intrahepatic cholangiocarcinoma, CD79α, HM47/A9

## Abstract

Hepatocellular carcinoma (HCC) and intrahepatic cholangiocarcinoma (ICC) are two major forms of primary liver cancer. The aim of this study was to investigate CD79α (HM47/A9) antibody expression patterns in normal liver, HCC and ICC samples. HM47/A9 expression was examined in tissues surrounding liver cancer in adults, 8-week embryos and 20-week embryos. In total, 82 cases of HCC, 31 cases of ICC and 11 cases of combined HCC and cholangiocarcinoma (cHCC-CC) were reviewed. HM47/A9 expression was observed as early as 8-week embryo liver tissue and exhibited focal granular cytoplasmic positivity, which was maintained throughout life. All 82 HCC cases demonstrated cytoplasmic granular positivity for HM47/A9, while no ICC cases were immunostained with HM47/A9. No CC components in cHCC-CC expressed the HM47/A9 antibody. A high number of HCC components in cHCC-CC showed positive staining for HM47/A9 [10/11 (90.9%)]. Our results suggest that HM47/A9 may be employed effectively to differentiate HCC from ICC.

## Introduction

Hepatocellular carcinoma (HCC) and intrahepatic cholangiocarcinoma (ICC) constitute two major forms of primary liver cancer. The two malignancies have different clinical and pathological features and prognosis, but in some cases histopathological overlap exists. Immunochemistry is thus required to facilitate differential diagnosis between HCC and ICC. To date, several antibodies, such as cytokeratin 19 (CK19), CD10, Hep Par 1, AFP, CA19-9, MOC31, glypican-3 and CEA, have been used to differentiate between the two malignant tumor types (1–3).

CD79α and CD79β belong to the Ig gene superfamily and contain one extracellular Ig-like domain, a transmembrane α-helical region and a cytoplasmic domain (3). These glycoproteins form a disulfide-linked heterodimer in the B-cell receptor (BCR) and pre-BCR complexex (4). The CD79α/β complex is critical to B-cell development, mediating signal transduction and promoting endocytosis of bound antigens for intracellular degradation and presentation to helper T cells (4). CD79α protein is present in B-cell follicles in lymph nodes, plasma cells and the majority of circulating B cells (5,6), but absent in brain, colon, kidney, liver, muscle, pancreas and placenta tissues. CD79α expression is not limited to B cells, as it is also detected in the normal early myeloid precursors and megakaryocytes (7). We (unpublished data) disclosed strong immunoreactivity of HM47/A9 with hepatocytes, but not with the bile canaliculus or interlobular bile duct. In the present study, we compared HM47/A9 antibody expression patterns in HCC and ICC.

## Materials and methods

### Cases selected for study

Normal adult livers were obtained from tissue surrounding the liver cancer. Eight-week embryo liver was acquired from a case of ruptured tubal pregnancy and the 20-week embryo liver sample was obtained from a perinatal mortality. We reviewed primary liver cancer cases between January 2002 and December 2012 in the People’s Liberation Army 152 Hospital (Henan, China), including 82 cases of HCC, 31 cases of ICC and 11 cases of combined HCC and cholangiocarcinoma (cHCC-CC), which were subjected to resection or puncture. All specimens were fixed in 10% neutral-buffered formalin, dehydrated in graded alcohol solutions, embedded in paraffin and cut into 4-μm-thick sections for hematoxylin and eosin staining, followed by visualization using light microscopy.

### Immunohistochemical analysis

Immunohistochemical staining was performed on formalin-fixed, paraffin-embedded tissue sections using the EnVision method. The primary antibodies employed included CD79α (HM47/A9), AFP (ZSA06), CK19 (A53-B/A2.26), MOC31 (MOC31), CA19-9 (TA888), CEA (Col-1) and Hepatocyte (OCH1E5). All antibodies were purchased from Maxin-Bio Co. (Fuzhou, China). Slides were counterstained with hematoxylin. To observe mallory hyaline bodies and globular hyaline bodies, slides were counterstained with eosin after immunostaining for CD79α.

## Results

### Clinical features

The 82 HCC patients included 48 males and 34 females with a median age of 46 years (range, 24–70 years). The 31 ICC patients included 16 males and 15 females with a median age of 51 years (range, 37–74 years). The 11 cases of cHCC-CC occurred in 6 males and 5 females with a median age of 48 years (range, 26–69 years).

### Histological, pathological and immunochemistry findings

**Figure 1:**
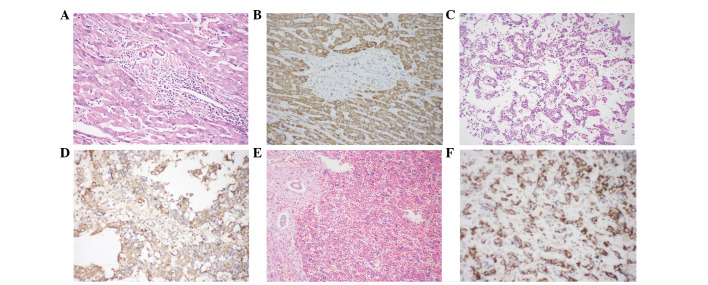
(A) Normal portal area. (B) Normal hepatocytes (99%) displaying cytoplasmic granular staining positive for HM47/A9. B cells in the portal area show membrane positivity for HM47/A9, while the bile canaliculus and interlobular bile duct are negative for HM47/A9. (C) Eight-week embryo liver. (D) Eight-week embryo hepatocytes (10%) exhibit granular positivity for HM47/A9. (E) 20-week embryo liver. (F) 20-week embryo hepatocytes (80%) show granular staining positive for HM47/A9. (A,B,D,F; magnification, ×120) (C and E; magnification ×60).

Normal hepatocytes (Fig. 1A) exhibited diffusely granular, cytoplasmic immunoreactivity to HM47/A9 (Fig. 1B). By contrast, no HM47/A9 positivity was observed in the bile canaliculus or interlobular bile duct (Fig. 1B).

In the 8-week embryo liver (Fig. 1C), ∼20% of hepatocytes displayed granular positivity for HM47/A9 (Fig. 1D). Hepatocytes of 20-week embryo liver (Fig. 1E) demonstrated diffuse immunoreactivity to HM47/A9 with a granular pattern in the cytoplasm (Fig. 1F), which was retained throughout life.

**Figure 2:**
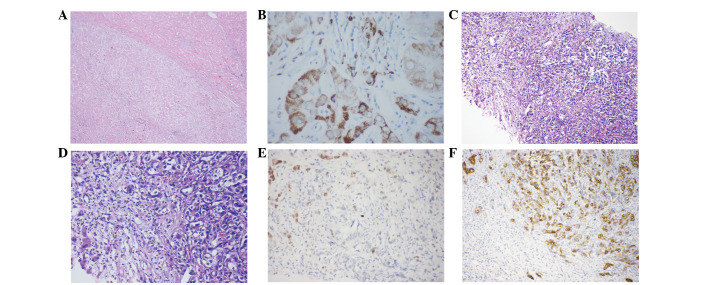
(A) HCC and surrounding liver tissue. Magnification, ×24. (B) HCC exhibiting (90%) positivity for HM47/A9. Magnification, ×240 (C and D) Low-power image depicting ICC and residual hepatocytes. Magnification, ×60 and ×120, respectively. (E) ICC negative for HM47/A9, hepatocytes positive for HM47/A9. Magnification, ×120. (F) ICC positive for CK19, hepatocytes negative for CK19. Magnification, ×120. HCC, hepatocellular carcinoma; ICC, intrahepatic cholangiocarcinoma.

HCC cells resembled hepatocytes (Fig. 2A). Some tumors had a plate-like pattern, while other HCC cells formed a pseudo glandular pattern. Tumor cells were round or oval-shaped with abundant granular eosinophilic cytoplasm and single, large central nuclei. Pale bodies and fatty changes were evident. HCC cells tested positive for Hepatocyte (79/82, 96.3%) and AFP (24/82, 29.3%) and negative for the CEA, CK19, CA19-9 and MOC31. All 82 HCC tumor cells exhibited diffuse granular positivity for HM47/A9 (Fig. 2B).

ICC cells were round or oval, and some were pleomorphic (Fig. 2C and D). Nuclei were small or large with more than one small nucleolus, while the cytoplasm was eosinophilic or vacuolated. Tumor cells formed a tubular gland or cord-like pattern, and all cases were negative for HM47/A9 (Fig. 2E). The tumor cells were positive for the CK19 (31/32, 96.9%) (Fig. 2F), CEA (7/10, 70%), CA19-9 (25/32, 78.1%) and MOC31 (26/32, 81.3%).

**Figure 3:**
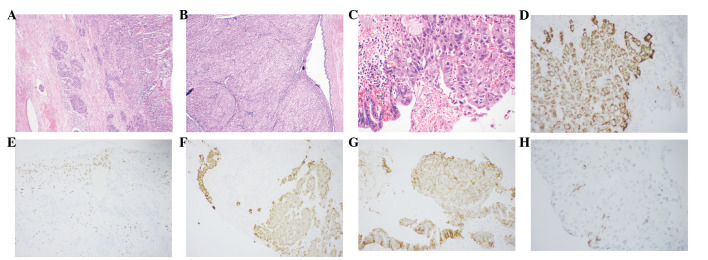
(A) Tumor thrombus. Magnification, ×24. (B and C) cHCC-CC invading the bile duct wall. Tumor cells displaying features of hepatocytes. (D) Tumor cells are immunoreactive for hepatocyte, while bile duct cells are negative for hepatocyte. Magnification, ×24 and ×120, respectively. (E) Inflammatory cells positive for pCEA, with no bile canaliculus observed between tumor cells. Magnification, ×60. (F and G) Tumor cells and bile duct cells positive for MOC31 and CK19, respectively. Magnification, ×60 and ×120, respesctively. (H) Tumor cells and bile duct cells negative for HM47/A9, B cells positive for HM47/A9 Magnification, ×120. cHCC-CC, combined HCC and cholangiocarcinoma.

Each of the CC parts in cHCC-CC [11/11 (100%)] did not express HM47/A9. The majority of HCC components in cHCC-CC [10/11 (90.9%)] exhibited positive staining for HM47/A9. The excluded case was in a 26-year-old female. These tumor cells demonstrated a similar histology to hepatocytes, with abundant eosinophilic or vacuolated cytoplasm and a centronucleus. Prominent nucleoli were observed in the nucleus. Tumor cells grew as entities and invaded the inter-lobular bile duct wall (Fig. 3A–C). Significant tumor thrombus in the hemal tube was evident (Fig. 3A). Tumor cells were immunoreactive for Hepatocyte (Fig. 3D), CK19 (Fig. 3F), CEA, CA19-9 and MOC31 (Fig. 3G), but negative for AFP and HM47/A9 (Fig. 3H). CEA (Fig. 3E) immunostaining data revealed no bile canaliculus between carcinoma cells.

**Figure 4:**
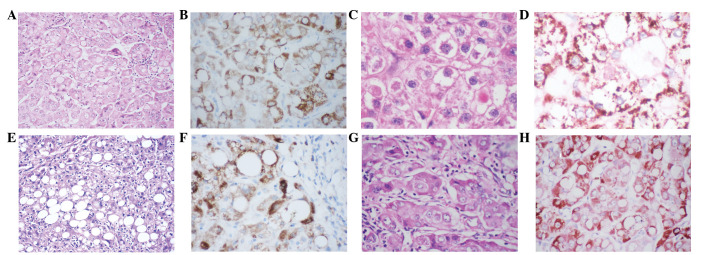
(A) Pale bodies, (C) mallory hyaline bodies, (E) fatty changes, and (G) globular hyaline bodies negative for HM47/A9 (B, D, F and H). (B and F) counterstained with hematoxylin; (D and H) counterstained with hematoxylin and eosin.(A and E; magnification, ×120) (B,C,D,F and G; magnification, ×240).

Pale bodies, mallory hyaline bodies, fatty degeneration and globular hyaline bodies were negative for HM47/A9 (Fig. 4).

## Discussion

CD79α, also known as Igα, is encoded by mouse B cell-specific gene 1 (mb-1) (3). The gene has been identified in pre-B and B cells, as well as thymocytes and peripheral blood T cells (3,8), but is absent in HeLa and kidney cells (3). In addition, no CD79α antigen has been detected in the liver, brain, colon, muscle or placenta tissue (5). However, we showed that hepatocytes exhibit strong cytoplasmic granular staining for HM47/A9, while the bile canaliculus and interlobular bile duct are negative for HM47/A9. Our results are inconsistent with previous reports (5), which may be attributed to our usage of the antibody clone. To date, JCM117 and HM-57 have been widely used by researchers to study CD79α expression (5), compared with 11E3, 11D10, SP18 and HM47/A9, which have seldom been employed. This discrepancy may be due to the different clones of CD79α antibodies.

CD79α expression precedes immunoglobulin heavy-chain gene rearrangement and CD20 expression, and disappears later than CD20 in the plasma cell (5). In our experiments, partial CD79α expression in human hepatocytes was observed in the 8-week embryo, and did not disappear in the adult. Based on these results, we conclude that HM47/A9 expression is activated with human hepatocyte development and maintained constitutively throughout human life. HM47/A9 may also be critically involved in human hepatocyte function. The liver is important in the metabolism of fat, carbohydrate and protein, intermediary metabolism as well as secretion. Further studies are required to elucidate the specific correlation between HM47/A9 and diverse liver functions.

We additionally compared HM47/A9 expression in hepatocytes from normal and diseased livers. As mentioned previously, HM47/A9 was expressed in hepatocytes, but not in the bile canaliculus or interlobular bile duct. In view of these findings, we hypothesized that HM47/A9 expression patterns differ among HCC, ICC and hepatic metastatic carcinoma. In this study, we observed that 82/82 HCC cases expressed HM47/A9, while no cases of ICC were positive for HM47/A9 (0/31). These results strongly support the utility of the HM47/A9 antibody in distinguishing HCC from ICC.

None of the 11 CC areas in cHCC-CC expressed HM47/A9. A majority of HCC components (10/11) in cHCC-CC showed positive staining for HM47/A9. The excluded case was a notable case of cHCC-CC, a rare subtype of primary liver carcinoma (9–11). Primary liver cancer cells exhibited features of HCC and invaded the interlobular bile duct. Tumor cells exhibited positivity for Hepatocyte, MOC31, CEA and CK19, and negativity for CD117 and AFP. Based on morphological and immunochemical results, diagnosis should be HCC with bile duct differentiation. Notably, however, these tumor cells were negative for HM47/A9. Earlier research has shown that cHCC-CC displays morphological and clinical similarities with cholangiocarcinoma and leads to poorer prognosis compared with pure HCC (12–14). However, other studies have reported similarities between cHCC-CC and HCC in terms of male/female ratio, status of hepatitis viral infection and serum AFP level (9,14). These two inconsistent findings may be attributed to different etiological roles according to the geographic situation or the existence of other carcinogenesis mechanisms (15,16). The case in this study displayed normal serum AFP level and no background chronic liver disease, with significant tumor thrombus. Our data support the findings of William RJ, who reported that cHCC-CC prognosis is similar to that of cholangiocarcinoma and worse than for pure HCC. Immunochemistry results disclosed tumor cell negativity for HM47/A9. As specified earlier, normal hepatocytes are positive for HM47/A9, while the bile canaliculus and interlobular bile duct are negative for HM47/A9. Thus, the poor prognosis of combined HCC and cholangiocarcinoma may be related to the loss of HM47/A9.

We additionally examined HM47/A9 expression in pale bodies, fatty degeneration, clear cell change, mallory hyaline bodies, intranuclear inclusion and glycogen. Notably, HM47/A9 granular expression was observed around pale bodies, fatty degeneration, clear cell change and mallory bodies, indicating that HM47/A9 is not a component of intermediate filaments, endoplasmic reticulum, glycogen and fat (17–20).

In conclusion, our results strongly suggest that HM47/A9 is an important protein component in hepatocytes that may be effectively employed to differentiate HCC from ICC. HCC with bile duct differentiation had poor prognosis, which may be related to the loss of HM47/A9.
